# Retraction: Extensive Pulmonary Embolism Following Mild COVID-19 Pneumonia

**DOI:** 10.7759/cureus.r132

**Published:** 2024-01-25

**Authors:** Ruba M Barnawi, Turki A Alsulami, Waleed A Alzahrani, Abdullah M Alsharif, Mohammed A Alsalaim, Ziyad A Alqazlan, Mohammed A Aljawi, Abdulaziz H Alghamdi, Mohammed A Alzubaidi, Zainab A Alqaysum, Hasan A Alabbad, Zied A Aljubour, Gadeer A Albannawi, Maryam M Alfaqih, Faisal Al-Hawaj

**Affiliations:** 1 Medicine, King Saud University, Riyadh, SAU; 2 Medicine, Debrecen University, Debrecen, HUN; 3 Medicine, Taif University, Taif, SAU; 4 Medicine, Almmarefa University, Riyadh, SAU; 5 Medicine, Najran University, Najran, SAU; 6 Medicine, Qassim University, Qassim, SAU; 7 Medicine, Taif Universty, Taif, SAU; 8 Medicine, Ibn Sina National College for Medical Studies, Jeddah, SAU; 9 Medicine, Jordan University of Science and Technology, Irbid, JOR; 10 Medicine, Imam Abdulrahman Bin Faisal University, Dammam, SAU; 11 Medicine, King Faisal Uneversity, Hofuf, SAU; 12 Medicine, Almareefa University, Riyadh, SAU; 13 Medicine, Umm Al-Qura University, Mecca, SAU

The Editors-in-Chief have retracted this article. Concerns were raised regarding the identity of the authors on this article. Specifically, Faisal Alhaway and Malak Shammari have stated that they were added to this article without their knowledge or approval. The identity of the other authors could also not be verified. As the appropriate authorship of this work cannot be established, the Editors-in-Chief no longer have confidence in the results and conclusions of this article.

